# Ocean Productivity May Predict Recruitment of the Rainbow Wrasse (*Coris julis*)

**DOI:** 10.1371/journal.pone.0165648

**Published:** 2016-11-08

**Authors:** Jorge Fontes, Brice Semmens, Jennifer E. Caselle, Ricardo S. Santos, Shree R. Prakya

**Affiliations:** 1 Marine and Environmental Sciences Centre (MARE), University of the Azores (IMAR/UAz), Dept. of Oceanography and Fisheries, 9901–862 Horta, Portugal; 2 Institute of Marine Research at the University of the Azores (IMAR/UAz), Dept. of Oceanography and Fisheries, 9901–862 Horta, Portugal; 3 Marine Biology Research Division, Scripps Institution of Oceanography, University of California San Diego, La Jolla, CA, 92023, United States of America; 4 Marine Science Institute, University of California Santa Barbara, Santa Barbara, CA 93106, United States of America; Leibniz Center for Tropical Marine Ecology, GERMANY

## Abstract

Predicting recruitment fluctuations of fish populations remains the Holy Grail of fisheries science. While previous work has linked recruitment of reef fish to environmental variables including temperature, the demonstration of a robust relationship with productivity remains elusive. Despite decades of research, empirical evidence to support this critical link remains limited. Here we identify a consistent and strong relationship between recruitment of a temperate wrasse *Coris julis*, from temperate reefs in the mid-Atlantic region, with Chlorophyll, over contrasting scales, across multiple years. Additionally, we find that the correlation between Chlorophyll and recruitment is not simply masking a temperature-recruitment relationship. Understanding the potential mechanisms underlying recruitment variability, particularly as it relates to changing climate and ocean regimes, is a critical first step towards characterizing species’ vulnerability to mismatches between pulsed planktonic production and early pelagic life stages.

## Introduction

For most of the 20th century, researchers have operated on the belief that year-class strength (and recruitment) in marine fishes is determined during the larval stage, when mortality rates are typically very high, with only a small portion of larval fish surviving to become adults [1]. This high, but variable, mortality results in coincidently variable and largely unpredictable recruitment [2]. Such intrinsic variability significantly impacts the population dynamics and demography of fish populations [3] and may confound management and conservation strategies. For this reason, since the birth of modern marine and fisheries ecology, scientists have devoted considerable attention to understanding and quantifying mortality in pelagic, early life stages of marine fish [4].

Much emphasis has been placed on understanding the processes governing recruitment variability and, ultimately, predicting annual recruitment. Fisheries ecologists have focused on the role of environmental processes occurring throughout the planktonic larval lives of marine fishes as determinants of survivorship and variability in recruitment (e.g. [5–8]). Such studies describe a range of environmental variables that may act independently or in concert to influence the survivorship of larvae. However, food supply and water temperature are believed to be the primary environmental determinants of marine fish larvae survival rates and recruitment variability [4,7–10].

Water temperature can be an important determinant of larval history as warmer temperatures typically lead to faster growth rates [4,9–12], shorter larval stage durations [10,12,13], and larger sizes-at-age [13], which may all influence larval survival rates [14]. Stronger competition for food [15], cannibalistic behavior [16] and faster yolk absorption with lower conversion efficiency of yolk to tissues [17] may also occur at elevated temperatures when metabolism is faster and therefore energy requirement is higher. Thus, faster growth due to elevated temperatures must be sustained with adequate food otherwise higher metabolic rates may increase susceptibility to starvation [4,9].

The premise that food limitation during the larval stage influences recruitment has been widely proposed [5,8,14,18,19] after it was first hypothesised by Hjort [20]. While expected in theory, relationships between early life stage survivorship and the physical environment and prey abundance have been difficult to establish empirically [4]. The idea that larval growth, survival and subsequent recruitment are dependent on the temporal matching of spawning periods with periods of high food availability is known as the match/mismatch hypothesis [18,19]. Evaluating the veracity of this hypothesis in marine fishes requires investigating the links between larval and planktonic population dynamics [7]. It was not until recently, owing to the development of remote sensing technology and availability of long data series, that this hypothesis was demonstrated in pelagic fishes (e.g [4,5,7,8]). However, evidence to support the match/mismatch hypothesis for reef fishes remains scarce (but see [21,22]). While a few studies found a weak correlation between temperature experienced during the larval stage and recruitment variability of tropical reef fishes (e.g. [11,13]), so far, most investigations have failed to establish a strong link between recruitment and larval food availability [11,13,23–26].

The aim of this paper was to investigate the link between seawater temperature, ocean productivity and recruitment variability of a common temperate reef fish, the rainbow wrasse (*Coris julis*; Linnaeus 1758), inter-annually and at two contrasting spatial scales.

We used satellite derived estimates of both sea surface temperature (SST) and chlorophyll (chl-a) as proxies for larval food availability and ocean temperature.

## Methods

### Ethics statement

This study was performed according to national Portuguese laws for the use of vertebrates in research. All procedures followed the guidelines for the use of fishes in research of the American Fisheries Society. No animals were manipulated or sacrificed. All data were obtained using under water visual fish counts with SCUBA.

### Study area and species

**Fig 1:**
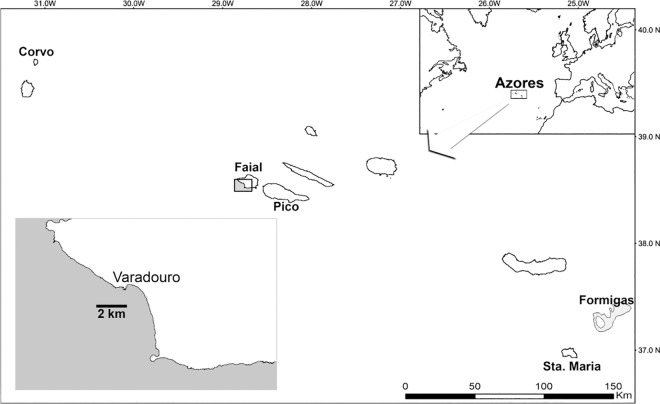
The Azores archipelago (NE Atlantic) and recruitment sampling sites. The Azores Archipelago (NE Atlantic) showing detail for Faial island and location of Varadouro.

The Azores, a nine island archipelago in the North Eastern Atlantic (Fig 1), are surrounded by deep water with limited shallow habitat. The Azores region is generally oligotrophic, and productivity is low. However, localized upwelling associated with island slopes and seamounts enhances local production [27]. The rainbow wrasse (*Coris julis*; Linnaeus 1758) is a small temperate, moderately short-lived (average 2 to 4 years) wrasse, and one of the most abundant reef fishes in the Azores [28]. The rainbow wrasse is a pelagic spawner with planktonic larvae. Larvae have a long pelagic larval dispersal phase lasting up to 46 days. Upon settlement, *C*. *julis* undergoes metamorphosis over a period of about 4 days, as indicated by a metamorphic band visible on the otoliths [29]. Spawning season in the Azores extends from June through August (Afonso & Morato, unpublished data). Although the larval diet of the rainbow wrasse is unknown, it is expected to be dominated by a diversity of copepod prey [30].

### Recruitment Surveys

We carried out recruitment surveys at two spatio-temporal scales: 1) Archipelago-wide surveys during a 3-year window, with the aim of classifying recruitment at multiple islands simultaneously, and 2) Location-specific surveys over a 6-year window to assess year-to-year recruitment variability following annual spawning. These two sets of data were analyzed separately, but with a similar aim: to assess the relationship between recruitment, temperature (SST) and productivity (Chlorophyll-a). Recruitment surveys consisted in underwater visual census (UVCs) of all settled recruits less than 50 mm (young-of-year YOY) within 20 x 1 m transects using SCUBA diving. Transects were placed over similar and dominant habitat, boulders covered in coralline and turf algae, at depths between 10 and 15 m. Transects were no less than 30 m apart.

#### Archipelago-wide annual surveys

From 2004 to 2006, we conducted annual recruitment surveys at four islands, Corvo (Western group), Faial, Pico (Central group) and Sta. Maria and one islet chain, Formigas (Eastern group). For detail and exact location see Fontes et al. [31]. Surveys occurred from late August to early September, in 2004 and 2005, and were completed at all sites within a 12-day period. In 2006, due to logistic constraints, surveys were performed, from late September to early October, within a 10-day window. Because we were limited to one cruise each year, we assume the density of young-of-year at the time of survey as our recruitment index. Researchers returned to the same sites every year using GPS and familiar topographic features. Five to six transects were surveyed at each site.

#### Location-specific surveys

Similarly, we surveyed YOY at Varadouro bay, Faial island, from 2003 to 2008. Surveys took place by the end of the recruitment season, late September to early October, and YOY were counted over four fixed transects, as detailed in Fontes et al. [31].

### SST and Chlorophyll-a measurements

We used satellite-based measurements of temperature (SST) and chlorophyll-a concentration as proxies for thermal environment and ocean productivity, respectively, for the areas of interest [26].

Ocean productivity and thermal environment was assessed using bi-weekly composites of MODIS Aqua Ocean Color Level-3 Chlorophyll-a concentrations (ocean color, mg/m3) and AVHRR Pathfinder Version-5 SST (c°) data, from a 50 by 50 km box extending 50 km south and 25 km to the west and east of each area where YOY were surveyed, with a spatial resolution for both data sets of 0.05 Deg. (approximately 4 km/pixel). Data were downloaded from NASA Goddard Space Flight Center (http://oceanwatch.pifsc.noaa.gov, last accessed on 9th Feb. 2015). For each 50 by 50 km box, we used the 14-day means of SST and Chl-a to calculate the average chlorophyll-a concentration and SST over a 90-day lagged period going back from the recruitment survey date. We used a 90-day lag because this period roughly overlapped with the larval stage of most cohorts that composed the YOY. Since survey dates were relatively variable among sites and years, lag periods were set from survey date to the previous 90 days, assuming this would increase the likelihood of measuring the environmental conditions experienced by the YOY over their larval stage.

### Statistical Analysis

#### Archipelago-wide annual surveys

Prior to model fitting, we normalized the YOY density data through log+1 transformation, and standardized (subtracted the mean and divided by the standard deviation) the SST and Chlorophyll-a data. We began by fitting a set of candidate models in a linear mixed effects framework, considering both islands (n = 5) and year (n = 3) as random effects, to explain the level of rainbow wrasse recruitment as a function of SST and Chlorophyll-a. Our intent was to use an information theoretic approach to evaluate relative support for different candidate models within this full parameterization using conditional Akaike's information criteria (cAIC–specific to mixed models; [32,33]). However, the models including random effects for year or site uniformly resulted in maximum likelihood estimates of random effect variance terms at ~zero (degenerate models). These results were not surprising given the small number of random effects in our sample, and the lack of clear separability of these effects within clusters. Mixed effect model estimates generate information theoretic results equivalent to models without the random effects included (when using marginal AIC reported by most mixed effects packages in R; cAIC is not possible to calculate with random effects variance terms are 0). As such, we opted to fit fixed effects models with both SST and Chl, and use AICc, a bias adjusted information criteria for small sample sizes, in order to evaluate relative model performance. The use of AICc is advisable for assessing candidate models using small amounts of data (as in our case), since the standard AIC calculation tends to select over-parameterized candidate models [34].

Our 4 candidate models (Table 1) differed only in their inclusion of the fixed continuous explanatory variables SST and Cholorophyll-a. We carried out model fitting using the package lme4 [35] in R, and calculated Akaike weights (i.e., normalized likelihood values; w) for each model. Additionally, we calculated relative variable importance values [W+; 34] for both explanatory variables across all candidate models. The relative importance values provide a rough measure of the relative explanatory power of predictor variables across all candidate models. Diagnostic plots for the model with the lowest AICc score are provided in S1 Fig.

**Table 1:** Candidate general linear models use to explain variability in recruitment based fixed effects of temperature and lagged chlorophyll-a concentrations. Relative model weights (a measure of relative model parsimony) are provided in the final column of the table.

Recruitment Model	K	log(L)	AICc	ΔAICc	w
β (Intercept only)	5	17.16	56.58	7.03	0.00
β1 + β2(Temp)	6	20.73	55.60	6.05	0.00
β1 + β2(ChlA)	6	13.85	49.55	-	0.97
β1 + β2(Temp) + β3(ChlA)	7	13.80	53.32	3.77	0.03

Number of effective parameters (K), log likelihood (log(L)), Akaike's bias-adjusted information criterion (AICc), and Akaike weights (w) are reported for each model.

Because SST and Chlorophyll-a were negatively correlated (rho = -0.58, p-value = 0.02), it is possible that the explanatory power of the variables was confounded. To further explore causal relationships between the two environmental variables and recruitment we used a causal modeling approach following Beaugrand and Kirby [36]. Simple causal modeling was based on the comparison of both simple and partial correlation coefficients. The partial correlation coefficient allows the relationship between two variables to be measured while removing the effect of a third variable (i.e. keeping its mean constant). Causal modeling was applied on triplets of variables using lagged Chlorophyll-a concentration, lagged SST and YOY density. The statistical causal link does not necessarily indicate a true causality of effect. However, the technique allowed the detection of spurious relationships that may be inferred by the ordinary coefficient of correlation [37], and provided an assessment of the explanatory power of the predictor variables considered. Linear regression analysis was also used to further investigate the relationship between the most significant variable selected by partial correlation analysis and recruitment.

#### Location-specific annual surveys

Since the data structure in the location scale study was not adequate to apply a linear mixed-effects modeling approach (absence of random term site), we used partial correlation analysis to investigate the causal relationship between YOY, lagged Chlorophyll-a concentration and lagged SST across years, following the same approach described above. As in the archipelago-wide scale, linear regression analysis was also used to investigate the relationship between the most significant variable selected by partial correlation analysis and YOY.

## Results

### Archipelago-wide annual recruitment

**Fig 2:**
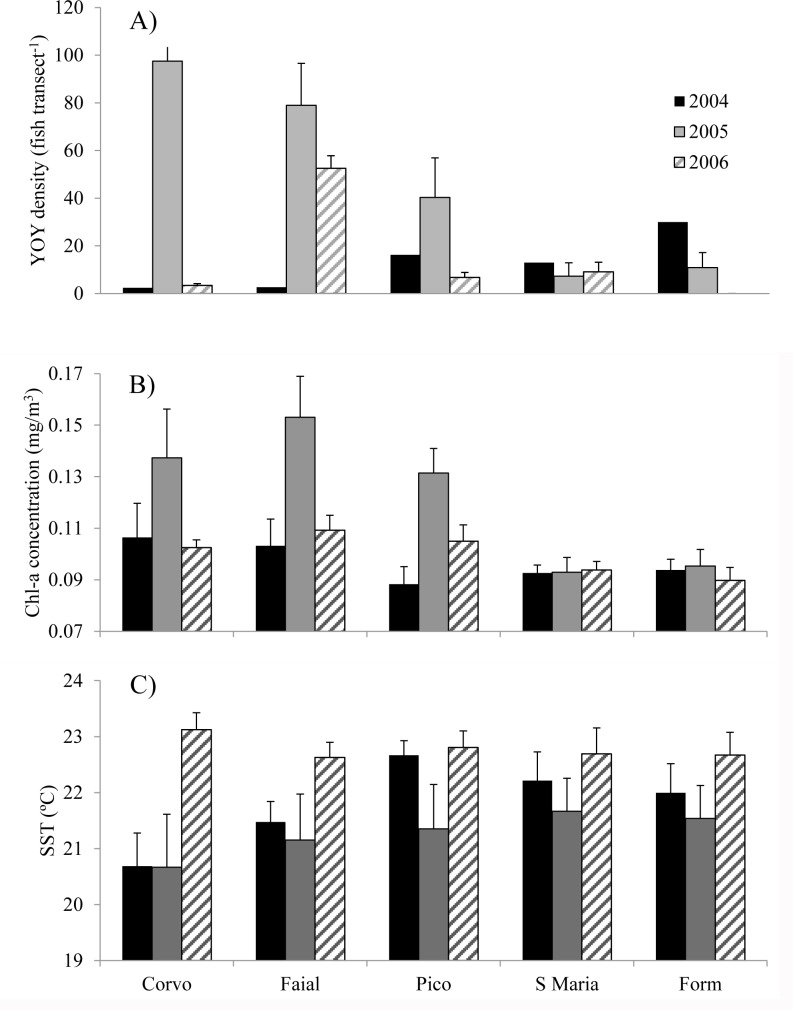
***Coris julis* young-of-the-year (YOY) density by site (panel A) and corresponding 90 day lagged averaged Chlorophyll-a concentration (panel B) and Sea Surface Temperature (panel C), from 2004 to 2006 across sites**. Values are means ± s.e.

Young of year (YOY) density across the Archipelago was highly variable within years compared to across years (Fig 2A). In 2004 we observed a steep gradient across the Archipelago, with increasing density from Corvo to Formigas and the inverse pattern in 2005. The maximum YOY density was observed at different sites each year and, in general, YOY abundance patterns were highly variable in space and time.

Mean lagged Chlorophyll-a concentrations (calculated over a 90 day period prior to surveys) were variable among years and island sites, with highest concentrations observed in 2005 at Corvo, Faial and Pico (Fig 2B). 2005 was also on average the coldest year, while 2006 was the warmest (Fig 2C). The lowest Chl-a concentrations were consistently observed at the southern and east most island sites, Sta. Maria and Formigas (Fig 2B).

The lowest AIC score of the four models was obtained for the model containing only average lagged Chl-a as an explanatory variable (Table 1).

**Fig 3:**
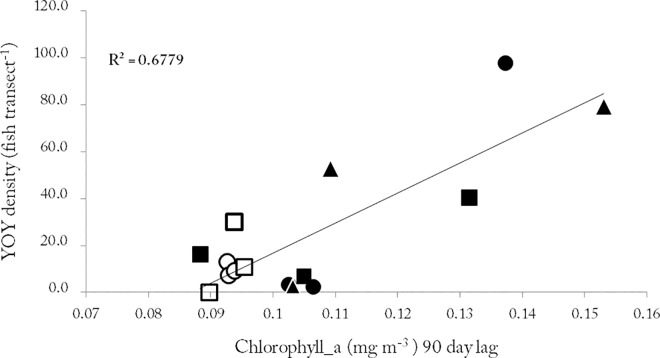
***Coris julis*.** Young-of-the-year (YOY) density *vs* 90 day Chlorophyll-a lagged averaged concentration and linear fit to the data, from 2004 to 2006, across all sites (black circles—Corvo, black triangles—Faial, black squares—Pico, white circles—Sta. Maria and white squares–Formigas).

However, given correlation between lagged Chl-a and SST, we carried out partial correlation coefficient analysis of lagged Chl-a and YOY density. With lagged SST influence removed, the partial correlation coefficient was 0.76 (p<0.01), whereas the partial correlation coefficient between lagged SST and YOY density, with lagged Chl-a influence eliminated, was -0.02 (p>0,05), indicating that the apparent relationship between lagged Chl-a concentration and recruitment was not masking that of lagged SST and recruitment. Linear regression analysis of lagged Chl-a concentration and recruitment showed that Chl-a explained almost 70% of inter-annual recruitment variation (R^2^ = 0.67; p>0.01) (Fig 3).

### Location-specific annual recruitment

**Fig 4:**
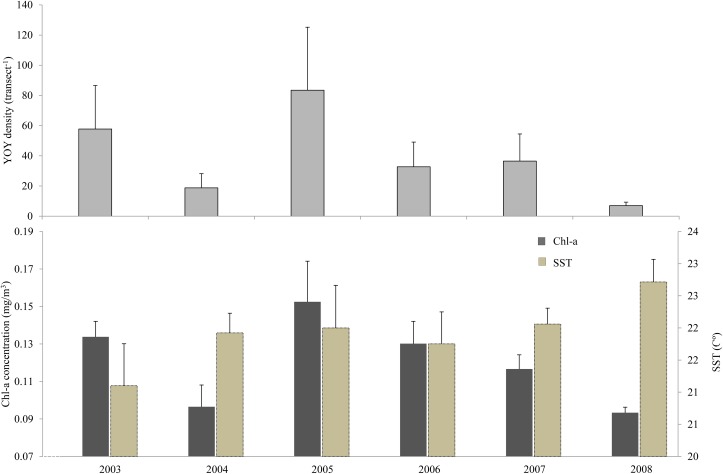
***Coris julis* young-of-the-year (YOY) density at Varadouro (panel A) and corresponding 90 day lagged averaged Chlorophyll-a concentration (light gray) and Sea Surface Temperature (dark gray), from 2003 to 2008 across sites (panel B)**. Values are means ± s.e.

Young of year (YOY) density was variable among years, with maximum density recorded in 2005, coincident with highest average lagged Chl-a concentration (Fig 4A and 4B). On the other hand, the lowest YOY densities were recorded in 2004 and 2008, when the lowest average lagged Chl-a concentration were measured. Except for 2005, Chl-a and SST typically followed a roughly inverse pattern (Fig 4).

Partial correlation analysis of lagged Chl-a, and YOY when controlling for lagged SST was 0.92 (p<0.01) whereas the partial correlation coefficient between SST and YOY, controlling for Chl-a, was -0.004 (p>0.05), indicating that the relationship between lagged Chl-a concentration and recruitment was much stronger than the correlation between lagged SST and YOY density.

**Fig 5:**
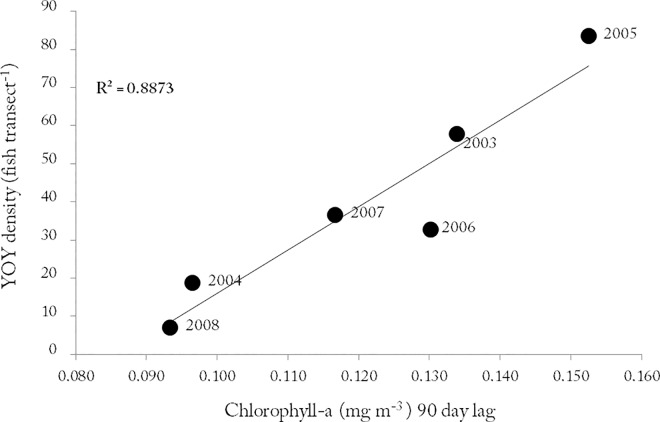
Linear fit of *Coris julis* young-of-the-year (YOY) density *vs* 90 day Chlorophyll-a lagged averaged concentration from Varadouro, Faial island, from 2003 to 2008.

Linear regression analysis of lagged Chl-a concentration and recruitment showed that Chl-a explained almost 90% of inter-annual recruitment variation (R^2^ = 0.88; p>0.01) (Fig 5).

## Discussion

Our results suggest that Chlorophyll concentrations during the larval phase were tightly linked to recruitment densities of rainbow wrasse, both locally and regionally. These results support the hypothesis that ocean productivity, as proxy for larval food abundance [8,25], is strongly correlated with recruitment success of one the most abundant reef fishes from the region. Although we analyzed a relatively shorter time series than most high impact studies published in the field (but see [8,22]), our results were consistent at two contrasting scales (across hundreds of Kms and locally, both across years, up to six recruitment seasons). Given the consistency of our results it is very unlikely that these results are spurious. Our use of a lag window of 90 days to develop predictor data was based on the pelagic life history of the species, and thus represents an appropriately conservative statistical approach to analyzing the data [34]. In other words, rather than using information criteria to evaluate a diverse number of potential lags (where the number of models would quickly eclipse the number of data points analyzed; [38]), we opted to identify, *a priori*, a single lag interval that best reflected our knowledge of the system-specific natural history. We recognize that a simple fixed lag interval is a gross over-simplification of the biological processes that mediate the relationship between productivity, planktonic survival and recruitment. However, even given this over-simplification, the strength of association between approximated ocean productivity and recruitment density suggests this relationship is readily observable even with coarse measures and limited data.

The link between ocean productivity, larval food abundance, fish larval survival and ultimately recruitment was first hypothesized more than a century ago by Hjort [20]. However, predicting recruitment fluctuations of commercially important fish populations remains the Holy Grail of fisheries science [39]. The few studies that successfully established an explicit link between ocean productivity or food abundance and fish recruitment focused almost exclusively on single species of commercial temperate pelagic fish stocks, where decadal-long time series of environmental variables, plankton abundance and recruitment are available [4–7,39]. The exception was Platt et al. [8] who analyzed 7 years of data from the eastern continental shelf of Nova Scotia, including two abnormally high recruitment events, and found that the survival of the larval haddock depended on the timing of the local spring bloom of phytoplankton. Simultaneously, multiple investigations have focused on the links between the pelagic larval environment and the recruitment of demersal reef fishes, both tropical and temperate (e.g. [11,23,25,26,40,41]). While these studies found some degree of correlation between recruitment and environmental variables, evidence of an explicit link between recruitment of reef fishes and ocean productivity remains elusive. Very few studies have reported empirical evidence to support the relationship between productivity in the larval environment and recruitment for reef fishes. Thresher et al. (1989) found that settlement pulses of the temperate reef fish *Heteroclinus sp*., from the large semiautonomous Storm Bay (Tasmania), matched lagged peaks of local phytoplankton production over three consecutive settlement seasons. However, this correlation became non-significant when years were pooled, reflecting poor correlation between year class strength and mean annual chlorophyll levels. Despite the small spatial scale and relatively short time series, Thresher et al. (1989) produced some of the first evidence to support the link between recruitment of reef fishes and ocean productivity before the advent of accessible high resolution remote sensing.

Hjort´s [20] seminal idea that recruitment is determined during early larval life in the plankton still prevails [39]. An important assumption of this theory is that primary production affects the quality and quantity of food available during the larval stage and therefore a lag between primary production and recruitment should exist, as shown in this study. An absence of a lag would suggest that the correlation is indirect [6]. Under this framework, the survival of fish larvae depends on their ability to encounter and ingest sufficient quantity of suitable prey in order to avoid starvation and to grow (e.g. [4,5,8]). The strong correlation between recruitment success and lagged ocean productivity, combined with previous evidence linking *C*. *julis* recruitment success with larval growth [29,42], supports the idea that recruitment variability was likely driven by fluctuations in larval food supply during the critical larval stage. In the Azores region the temporal window for successful optimal larval development is likely related to the spring bloom that provides most of the annual food supply and mediates survival of copepods in the plankton [16]. Thus, variability in the magnitude and timing of the spring bloom is likely to have a significant impact on survival of late spring and summer larvae, like the rainbow wrasse.

Even though we didn’t find evidence of a strong link between temperature and recruitment, this environmental variable may indirectly affect larval survival and recruitment, by modifying plankton abundance, composition and distribution or by influencing larval metabolism and growth. For example Beaugrand et al. [5] found that rising temperature in the North Sea since the mid-1980s had a negative impact on cod recruitment by modifying prey size, timing of plankton blooms, prey abundance and distribution. It was suggested that the combination of decreased prey abundance and the increase in cod metabolism, expected in warmer years, likely reduced early stages survival rates and led to poor recruitment. Similar arguments have been suggested to explain recruitment variations in tropical reef fishes. For example Sponaugle et al. [10], despite having found a positive correlation between water temperature and larval growth of the bluehead wrasse from the upper Florida Keys, reported quite variable recruitment success among warm-water cohorts. They speculated that such variability was, in part, a consequence of the intrinsic difficulty in sustaining high growth rates in warm water, particularly when food was limited. In summary, the combination of warm water and adequate food supply should result in high survival rates and potentially large recruitment events, while a detrimental effect is to be expected in warm water conditions when food is limited [4], which is arguably the case in this oligotrophic region [27].

The average temperature fluctuations observed in this study were well within the optimal temperature range for the rainbow wrasse considering its wide latitudinal distribution ranging from Sweden to south of Cape Lopez, Gabon, including the Azores, Madeira and the Canary Islands [43]. A wide thermal tolerance and relatively narrow water temperature variation during the study period, about 2°C, may explain why water temperature was found to have little correlation with recruitment.

It is interesting to note that our findings are more in alignment with results from recruitment dynamics studies from northern pelagic fish stocks, such as cod or haddock, than with other investigations from temperate upwelling regions. In these coastal temperate upwelling systems, recruitment has been difficult to predict probably owing to the complex interaction of multiple physical and environmental variables, each showing very large temporal and spatial variation (e.g. [23,26,41]).

The findings presented here contribute to the growing body of literature linking characteristics of the larval environment with recruitment patterns. As this and other such studies continue to elucidate environment-recruitment dynamics, we anticipate that future fisheries management decisions will be bolstered by the ability to predict future recruitment using remotely measured environmental variables. However, it is important to note that the importance of the underlying factors may change with time [44], especially in light of rapid climate change. Given that climate changes can lead to a mismatch between trophic levels by modifying functional groups in the marine pelagic community [45], recruitment success of fishes, such as the rainbow wrasse, that depend on the matching between larval production and their food may be at risk in a changing ocean.

## Supporting Information

S1 Data azores50K. (XLSX)

S1 Fig Diagnostics associated with the model regressing rainbow wrasse recruitment and cholorophyll-a. (DOCX)
