# Supplementary material for: Ocean Productivity May Predict Recruitment of the Rainbow Wrasse (Coris julis)
Source: PLoS One. 2016 Nov 8;11(11):e0165648. doi: 10.1371/journal.pone.0165648 (PMC5100946; doi:10.1371/journal.pone.0165648)

**Supporting Information File S1**

**Ocean productivity predicts recruitment of the rainbow wrasse (*Coris julis*).**

J. Fontes, B. Semmens, J. E. Caselle, R. S. Santos, S. R. Prakya

**S1 Fig. Diagnostics associated with the model regressing rainbow wrasse recruitment and cholorophyll-a**


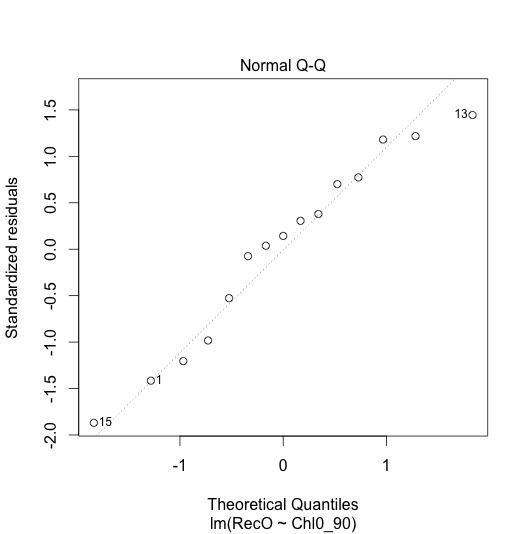


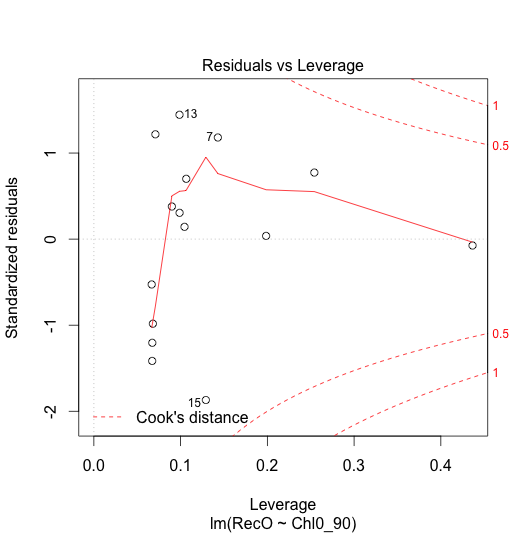

Supplement: S1 Fig — (DOCX) [file pone.0165648.s002.docx]
